# Evaluation of the factors affecting the maximum standardized uptake value of metastatic lymph nodes in different histological types of non-small cell lung cancer on PET-CT

**DOI:** 10.1186/s12890-015-0014-2

**Published:** 2015-03-08

**Authors:** Yuehong Wang, Shanni Ma, Mengjie Dong, Yake Yao, Kanfeng Liu, Jianying Zhou

**Affiliations:** Department of Respiratory Medicine, the First Affiliated Hospital, College of Medicine, Zhejiang University, 79 Qingchun Road, Hangzhou, China; Department of Nuclear Medicine, the First Affiliated Hospital, College of Medicine, Zhejiang University, 79 Qingchun Road, Hangzhou, China

**Keywords:** PET-CT, Lymph node metastases, Squamous cell carcinoma, Adenocarcinoma, Sensitivity, Specificity

## Abstract

**Background:**

To evaluate the factors affecting the maximum standardized uptake value (SUVmax) of metastatic lymph nodes in different histological types of non-small cell lung cancer (NSCLC) on integrated positron emission tomography and computed tomography (PET-CT).

**Methods:**

This was a retrospective, single-institution review of 122 patients with pathologically proven NSCLC who had PET-CT scanning at the same center. Lymph node metastases were pathologically confirmed in tissue specimens from surgical patients. Statistical evaluation of PET-CT results was performed on a per-nodal-station basis.

**Results:**

The tumor SUVmax of squamous cell carcinoma (SCC) (11.0 ± 4.1) was higher than that of adenocarcinoma (AC) (7.4 ± 4.4) (P < 0.01), however, the SUVmax of the metastatic lymph nodes did not differ between the SCC (4.6 ± 3.1) and AC groups(3.6 ± 2.5) (P = 0.221). The SUVmax of metastatic lymph nodes was positively correlated with lymph node size but not with the primary tumor SUVmax, primary tumor size, tumor location and tumor differentiation. The frequency of a SUVmax of lymph nodes ≥2.5 was 44%, 80%,100% in SCC group and 39%, 59%, 90% in AC group when the short-axis diameter of metastatic lymph node was <10 mm, 10–15 mm, and > 15 mm, respectively. The low sensitivity for metastatic lymph nodes on PET-CT was increased when the SUVmax cut-off for malignancy was considered to be above the normal background compared with that when the SUVmax cut-off was above 2.5.

**Conclusions:**

There was no difference in the SUVmax of metastatic lymph nodes in the SCC and AC groups. The SUVmax of metastatic lymph nodes was positively correlated with metastatic lymph node size. There was a high false negative rate if lymph nodes with a short-axis diameter less than 10 mm and a extremely low false negative rate if lymph nodes with a short-axis diameter higher than 15 mm. Although an increased sensitivity may be achieved by decreasing the SUVmax cut-off, invasive staging may still be required for negative lymph nodes due to the lower sensitivity of PET-CT in both SCC and AC.

## Background

Lung cancer is the most commonly diagnosed cancer and the leading cause of cancer-related deaths worldwide [1]. Non-small cell lung cancer (NSCLC), which mainly includes squamous cell carcinoma (SCC) and adenocarcinoma (AC), accounts for 80% of lung cancer cases. Accurate lymph node (LN) staging of NSCLC is one of the most important factors in the selection of appropriate treatment and in the determination of patient prognosis. Incorrect staging of NSCLC can result in unnecessary thoracotomies and early local or distant relapse after surgery [2,3].

Since 2001, integrated positron emission tomography and computed tomography (PET-CT), which provides both morphological and metabolic information, has been increasingly used for assessing lymph node metastases in patients with NSCLC because of its advantages of safety and accuracy. The rationale for using FDG-PET in oncology is its ability to measure the increased glucose metabolism of tumor cells. A maximum standardized uptake value (SUVmax) greater than 2.5 is usually used as a cut-off value for malignancy [4]. Recent studies have shown that PET-CT can provide high specificity in LN staging in NSCLC. However, the sensitivity of PET-CT in mediastinal LN staging varies from 40–86.3% [5-12]; thus, there is a significant number of false-positive and false-negative findings in LN staging of lung cancer. The major reasons for the false-positive and false-negative findings in LN staging are lymph node involvement resulting from inflammatory diseases and microscopic metastases [5-12]. The ability of PET-CT to directly assess each lymph node station is limited; therefore, other approaches need to be explored to increase the accuracy of LN staging of NSCLC.

Some relevant studies have shown that primary tumors with different histological types produce different SUVmax values on integrated PET-CT. SCC exhibited higher SUVmax values than AC [13-15]. However, it still remains unclear whether the histological type of NSCLC affects the assessment of thoracic metastatic lymph nodes and which other factors may influence the SUVmax of metastatic lymph nodes on PET-CT. Thus, the purpose of this study was to evaluate the factors that affect the detection of metastatic lymph nodes in different NSCLC types (SCC and AC) by comparing pre-operative PET-CT scan results with corresponding post-operative pathological findings.

## Methods

### Subjects

This study retrospectively reviewed 122 consecutive patients with pathologically proven NSCLC (SCC and AC) who underwent surgery and had integrated PET-CT scanning between February 2008 and April 2013 at First Affiliated Hospital, College of Medicine, Zhejiang University. This retrospective study was approved by the review board of First Affiliated Hospital, College of Medicine, Zhejiang University,which waived the requirement for patients’ informed consent.

In addition to integrated PET-CT, all patients underwent a conventional diagnostic workup, including a thorough history, physical examination, laboratory tests and contrast-enhanced chest CT, to evaluate the condition of the brain, bones and abdomen.

Patients who received induced chemotherapy and/or radiation therapy, patients who had diabetes mellitus or a high serum glucose level (greater than 150 mg/dL) at the time of PET-CT examination and patients with histological types other than SCC and AC were excluded.

We retrospectively collected data on the enrolled patients and prospectively compiled an electronic database.

### Integrated PET-CT

All PET-CT images were obtained using a combined PET-CT scanner (Biograph Sensation 16, LSO 39 ring, Siemens Medical, Erlangen, Germany). All patients were asked to fast for at least 6 h before the examination and then received an intravenous injection of FDG at 5.5-7.4 MBq (0.15-0.20 mCi)/kg of body weight. After 1 h, the patients were scanned from the head to mid-thigh using an integrated PET-CT system. First, a 16-section multi-detector row CT scan was performed at 120 kV and 50 mA. Then, with a tube rotation time of 0.5 seconds, a 2.5-mm-thick section was matched to the PET section thickness, followed by a three-dimensional PET with the patient in the same supine position. The PET emission scan covered the region from the subcranium to the mid-thigh. The brain scan required another bed position, and the acquisition time was 2 minutes per bed position. Attenuation correction was based on CT. The PET images were iteratively reconstructed using ordered subset Syngo Speaking software (Wizard Workstation; Siemens Medical). PET, CT and fused PET/CT images were generated and reviewed on a computer, and co-registered images were displayed on a workstation.

Two experienced nuclear medicine physicians who were aware of the clinical and stand-alone contrast-enhanced CT results, but were blinded to the histological findings, evaluated the PET-CT data side by side in consensus. A lesion with increased FDG uptake in three planes compared with background or with a SUVmax above 2.5 on PET scan was classified as malignant. Then, the SUVmax and diameter of the primary tumors and short-axis diameter of lymph nodes were measured and collected.

### Surgery and histopathology

A total of 122 patients underwent surgical resections and nodal dissections at First Affiliated Hospital, College of Medicine, Zhejiang University. Pulmonary resections included pneumonectomy (n = 2), bilobectomy (n = 14), lobectomy (n = 88) and segmentectomy (n = 18). All resected tumor specimens were examined and classified based on the World Health Organization (WHO) classification. The dissected lymph nodes were stained by hematoxylin and eosin and then histologically examined. These pathological examinations were all performed by experienced pulmonary pathologists at the same hospital. By retrospectively reviewing the pathological results of the enrolled patients, we collected information on the diameter and SUVmax of the primary tumors, short-axis diameter of thoracic lymph nodes, pathological type, tumor location and tumor differentiation.

### Data analysis

Continuous data are reported as medians, variances and ranges, while categorical data are reported as counts and percentages. The sensitivity, specificity and accuracy of integrated PET-CT in assessing lymph node metastases were determined based on the histological results as the reference standard. Correlation between the SUVmax of metastatic lymph nodes and potential covariates was performed using a linear regression model of general evaluation equation, and the chi-square test was used for categorical data (sensitivity, specificity and accuracy). All P values were two-sided, and a P value <0.05 was considered statistically significant. All analyses were conducted using the SPSS 16.0 software package.

## Results

### Subjects

The study population included 122 patients who underwent surgery; SCC and AC were the final diagnoses in 41 (33.6%) and 81 (66.4%) patients, respectively. The median ages of the SCC and AC patients were 61.9 ± 11.2 and 61.5 ± 10.7 years, respectively. A total of 511 nodal stations were evaluated, including 195 in the SCC group and 316 in the AC group. A total of 27 of 195 SCC nodal stations and 80 of 316 AC nodal stations were pathologically positive for malignancy.

### Characteristics of tumors and lymph nodes according to histological type

The characteristics of primary tumors and lymph nodes are summarized in Table 1 according to histological type. Among all enrolled patients, the mean SCC and AC diameters were 39.5 ± 10.2 and 25.5 ± 8.9 mm, respectively (P < 0.01), and the mean SUVmax of SCCs and ACs was 11.0 ± 4.1 and 7.4 ± 4.4, respectively (P < 0.01). The SUVmax of the metastatic lymph nodes in the SCC and AC groups was 4.6 ± 3.1 and 3.6 ± 2.5, respectively (P = 0.221). The diameter of the metastatic lymph nodes was 11.9 ± 3.7 mm and 12.7 ± 4.5 mm in the SCC and AC groups, respectively (P = 0.877).

**Table 1:** **Characteristics of primary tumors and lymph nodes in NSCLC**

**Parameter**	**SCC(n = 41)**	**AC(n = 81)**	**P**
Tumour diameter(mm ± SD)	39.5 ± 10.2	25.5 ± 8.9	<0.001
Tumour SUVmax(±SD)	11.0 ± 4.1	7.4 ± 4.4	<0.001
Metastastic lymph nodes diameter (mm ± SD)	11.9 ± 3.7	12.7 ± 4.5	0.635
Metastastic lymph nodes SUVmax (±SD)	4.6 ± 3.1	3.6 ± 2.5	0.221
Location of tumor			
Central	28	7	
Peripheral	13	74	
Degree of differentiation			
High	8	7	
Moderately	13	15	
Poorly	20	59	

### Correlation between the SUVmax of metastatic lymph nodes and potential covariates

The factors that may affect the SUVmax of metastatic lymph nodes in NSCLC patients have not been determined. We analyzed the correlation between the SUVmax of metastatic lymph nodes and other factors such as the primary tumor SUVmax and size, metastatic lymph node size, primary tumor location, tumor differentiation and histological type using linear regression model of general evaluation equation (Table 2). Only lymph node size was significantly related to the SUVmax of metastatic lymph nodes (P <0.0001). The SUVmax and size of the primary tumor, the location of the primary tumor, tumor differentiation and histological type had no correlation with the SUVmax of metastatic lymph nodes. Table 3 shows the relationship between the metastatic lymph node size and the frequency of a SUVmax of lymph nodes ≥ 2.5. Our study demonstrates that the frequency of a SUVmax of lymph nodes ≥2.5 was 44%, 80%,100% in SCC group and 39%, 59%, 90% in AC group when the metastatic lymph size was <10 mm, 10–15 mm, and >15 mm, respectively.

**Table 2:** **The associations of tumor size, tumor SUVmax, tumor differentiation, tumor location, lymph node size and tumor pathological type with metastatic lymph node SUVmax were analyzed, using linear regression model of general evaluation equation (GEE)**

**Variable**	**Class**	**β**	**se**	**P**
Tumor size		0.03	0.09	0.7069
Tumor SUVmax		0.03	0.03	0.3429
Tumor differentiation	High	Ref	--	--
	Median	−0.04	0.62	0.9526
	Low	−0.48	0.58	0.4035
Tumor location	Central	Ref	--	--
	Peripheral	0.05	0.35	0.8917
Lymph node size		0.17	0.04	<0.0001
Pathological type	Squamous cancer	Ref	--	--
	Adenocarcinoma	−0.16	0.4	0.6872

**Table 3:** **Relationship between lymph node size and frequency of SUVmax ≥ 2.5**

**Range of lymph node size**	**Frequency of SUVmax ≥ 2.5**	
	**SCC**	**AC**
<10 mm	44%	39%
10 mm≤, ≤15 mm	80%	59%
15 mm<	100%	90%

### Sensitivity, specificity and accuracy of PET-CT based on different SUVmax cut-offs

For criterion 1, the lymph nodes were deemed malignant when their SUVmax was higher than the normal background. According to criterion 1, in the SCC group, 14 nodal stations were false-negatives, and 13 nodal stations were true-positives; in the AC group, 46 nodal stations were false-negatives, and 34 nodal stations were true-positives. The sensitivity, specificity and accuracy were 48.1%, 88.1% and 82.6%, respectively, in the SCC group (Table 4) and 57.5%, 95.9% and 86.3%, respectively, in the AC group (Table 5) based on a per-nodal analysis.

**Table 4:** **Contingency table for PET-CT in identifying lymph node metastases in SCC group**

	**Criterion 1**			**Criterion 2**		
	**Pathological LN positive(+)**	**Pathological LN negative(−)**	**Total**	**Pathological LN positive(+)**	**Pathological LN negative(−)**	**Total**
**PET-CT(+)**	13	12	25	10*	16	26
**PET-CT(−)**	14	156	170	17	152	169
**Total**	27	168	195	27	168	195

Criterion 1: Sensitivity: 48.1%; Specificity: 88.1%; Accuracy: 82.6%.

Criterion 2: Sensitivity: 37.0%*; Specificity: 90.5%; Accuracy: 83.1%.

*Means the difference in the sensitivity was significant (P < 0.01) between criterion 1 and criterion 2 in SCC group.

**Table 5:** **Contingency table for PET-CT in identifying lymph node metastases in AC group**

	**Criterion 1**			**Criterion 2**		
	**Pathological LN positive(+)**	**Pathological LN negative(−)**	**Total**	**Pathological LN positive(+)**	**Pathological LN negative(−)**	**Total**
**PET-CT(+)**	46	10	56	32*	9	41
**PET-CT(−)**	34	232	266	48	233	281
**Total**	80	242	322	80	242	322

Criterion 1: Sensitivity: 57.5%; Specificity: 95.9%; Accuracy: 86.3%.

Criterion 2: Sensitivity: 40.0%*; Specificity: 96.3%; Accuracy: 82.3%.

*Means the difference in the sensitivity was significant (P < 0.01) between criterion 1 and criterion 2 in AC group.

For criterion 2, the lymph nodes were deemed malignant when their SUVmax was above 2.5. The sensitivity, specificity and accuracy were 37.0%, 90.5% and 83.1%, respectively, in the SCC group (Table 4) and 40.0%, 96.3% and 82.3%, respectively, in the AC group (Table 5) according to criterion 2. The difference in the specificity and accuracy in both the SCC and AC groups was not significant between criterion 1 and criterion 2. However, the sensitivity was higher when criterion 1 was used compared with criterion 2 (P < 0.01).

## Discussion

In keeping with published reports, the SUVmax of the primary tumor was significantly higher in the SCC group (11.0 ± 4.1) than in the AC group (7.4 ± 4.4) [16,17]. Although the uptake mechanism and biochemical pathways of FDG are not completely understood, previous studies showed that glucose transporters (Gluts) are important factors that influence FDG uptake by malignant tumors and that Glut-1 is the principal subtype in NSCLC. It was reported that the degree of Glut-1 expression in SCC is higher than that in AC, which may partly explain why the SUVmax of SCC is higher than that of AC [18-20]. It also has been reported that the SUVmax of NSCLC is positively correlated with tumor size. In our study, we found that the primary tumor size of SCC was significantly greater than that of AC, which could be another cause of the higher tumor SUVmax in the SCC group.

Although it is indicated that the SUVmax of SCC is higher than that of AC, it still remains unclear whether the SUVmax of metastatic lymph nodes in SCC patients is also higher than that in AC patients. Our study showed that there was no difference in the SUVmax of metastatic lymph nodes between the SCC (4.6 ± 3.1) and AC groups (3.6 ± 2.5). This result demonstrated that the histological type of NSCLC does not affect the SUVmax of metastatic lymph nodes even though it influences the SUVmax of the primary tumor. In this study, multivariable linear regression revealed that the SUVmax of metastatic lymph nodes had a positive correlation with lymph node size, but the SUVmax and size of the primary tumor, location of the primary tumor, and tumor differentiation had no influence on the SUVmax of metastatic lymph nodes. This result indicates that the size of the lymph nodes may be the key factor that affects the SUVmax of metastatic lymph nodes. Our study also showed that when the short-axis diameter of the lymph node was <10 mm, only 44% of metastatic lymph nodes in SCC group, 39% of metastatic lymph nodes in AC group had a SUVmax higher than 2.5, whereas about 100% of metastatic lymph node in SCC group and 90% of metastatic lymph node in AC group had a SUVmax higher than 2.5 when the short-axis diameter of the lymph node was >15 mm. Bille et al. [21] analyzed the factors associated with metastatic lymph node detection in patients with AC vs. SCC, and they found that in the AC group, the mean diameter of false-negative lymph nodes was 7 ± 2.5 mm, compared with the 12.5 ± 4 mm diameter of true-positive lymph nodes. In the SCC group, the mean diameter of false-negative lymph nodes was 7.4 ± 2.8 mm, compared with the 14.7 ± 6 mm diameter of true-positive lymph nodes. Regarding nodal size, the PET-CT sensitivity in detecting malignant involvement was 32.4% in nodes <10 mm and 85.3% in nodes ≥10 mm [11]. According to the above results, we may have to do with the fact that there is a higher false-negative rate if the diameter of the lymph node is <10 mm, and a extremely low false-negative rate if the diameter of the lymph node is >15 mm in both SCC and AC. Nambu et al. [22] found that lymph node metastases were more commonly observed in NSCLC cases with a higher tumor SUVmax. The frequency of lymph node metastases was 70% in NSCLC patients with a tumor SUVmax greater than 12, whereas no lymph node metastases were found in NSCLC patients with a tumor SUVmax less than 2.5. These results suggest that lung cancer patients exhibiting a high tumor SUVmax may have a high risk for lymph node metastases. Similar results have also been reported in other studies [4,23]. However, our study revealed that there is no correlation between the SUVmax of the primary tumor and the SUVmax of the metastatic lymph node, which may due to the smaller sample size in our study. In clinical practice, it is challenging to distinguish metastatic lymph nodes from benign lymph nodes, especially for those lymph nodes with a short-axis diameter of less than 10 mm, which are always classified as negative for metastasis before surgery. According to the studies mentioned above, we believe that those lymph nodes with a short-axis diameter less than 10 mm should not be classified as negative despite having a SUVmax score of less than 2.5. If those small size lymph nodes have a higher primary tumor SUVmax, there is a high probability of metastasis, and further invasive procedures such as EBUS-TBNA, mediastinoscopy or VATS may be considered.

Previous studies indicated that PET-CT has a low sensitivity and a relatively high specificity for detecting thoracic lymph node metastases. However, to date, the ideal SUVmax cut-off for distinguishing malignant from benign thoracic lymph nodes has not been determined. Most studies have defined a SUVmax of 2.5 as the upper limit of normal lymph nodes. However, this cut-off is purely arbitrary. Some previous studies also considered positive mediastinal lymph nodes to be nodes that exhibit focally increased 18-FDG uptake above the normal background activity. In our study, we found that using the SUVmax cut-off of 2.5 resulted in a considerable number of false negatives for pathologically metastatic lymph nodes. In order to determine whether the sensitivity could be improved by adjusting the criteria, we compared the sensitivity, specificity and accuracy of PET-CT in detecting metastatic lymph nodes using two different SUVmax cut-offs.

Our study showed that there was no significant difference in the specificity and accuracy of the two criteria in either the SCC or AC group. The sensitivity in the AC and SCC groups detected using criterion 1 was significantly higher than that using criterion 2. The different SUVmax cut-offs significantly affected the sensitivity but not the specificity. Therefore, we suppose that a SUVmax cut-off value of 2.5 for detecting metastatic lymph nodes may not be optimal for sensitivity because lymph nodes with a slight SUVmax increase may be associated with a missed diagnosis. Bille et al. assessed the sensitivity, specificity and accuracy of PET-CT for detecting lymph node metastasis in different histological types of NSCLC; lymph nodes were deemed positive for metastatic spread if they exhibited focally increased FDG uptake that was higher than the normal background activity [22]. The study showed that the sensitivity, specificity and accuracy of PET-CT were 53.8, 91.5 and 79.1%, respectively, in the AC group and 87.5, 81.8 and 83.5%, respectively, in the SCC group. Although the sensitivity may be increased when lymph nodes are defined as malignant when their SUVmax is higher than the background, this scenario will also increase the possibility of a false positive result. Therefore, the results of this preliminary study need to be confirmed.

Our study has several limitations. First, this study was retrospective in design, and there may have been bias. Second, this study examined a limited number of patients, and the study population consisted of only patients who underwent surgery; thus, a definitive conclusion cannot be drawn.

## Conclusions

In conclusion, there is no difference in SUVmax of thoracic metastatic lymph nodes between SCC group and AC group, and the SUVmax of thoracic metastatic lymph nodes is mainly related to their size in both SCC and AC patients. There was a high false negative rate if lymph nodes with a short-axis diameter less than 10 mm and a low false negative rate if lymph nodes with a short-axis diameter higher than 15 mm. There was a low sensitivity and high specificity and accuracy of PET-CT in assessing malignant thoracic lymph nodes in both SCC and AC patients; the sensitivity may increased when the SUVmax cut-off for distinguishing malignant thoracic lymph nodes was considered to be above the normal background. The results of this study need to be confirmed by prospective studies with larger sample sizes.

## Abbreviations

PET-CT: Integrated positron emission tomography and computed tomography
SUVmax: Maximum standardized uptake value
NSCLC: Non-small cell lung cancer
SCC: Squamous cell carcinoma
AC: Adenocarcinoma
LN: Lymph node
